# Functional Portrait of Irf1 (Orf19.217), a Regulator of Morphogenesis and Iron Homeostasis in *Candida albicans*

**DOI:** 10.3389/fcimb.2022.960884

**Published:** 2022-08-08

**Authors:** Lasse van Wijlick, Sadri Znaidi, Arturo Hernández-Cervantes, Virginia Basso, Sophie Bachellier-Bassi, Christophe d’Enfert

**Affiliations:** ^1^ Institut Pasteur, Université Paris Cité, INRAE USC2019, Unité Biologie et Pathogénicité Fongiques, Paris, France; ^2^ Institut Pasteur de Tunis, Laboratoire de Microbiologie Moléculaire, Vaccinologie et Développement Biotechnologique, Tunis-Belvédère, Tunisia

**Keywords:** *Candida albicans*, iron homeostasis, trace metals, RNA-Seq, ChIP-Seq, Orf19.217/Irf1, hyphal morphogenesis, commensalism

## Abstract

The alternate growth of *Candida albicans* between a unicellular yeast form and a multicellular hyphal form is crucial for its ability to cause disease. Interestingly, both morphological forms support distinct functions during proliferation in the human host. We previously identified *ORF19.217* (C2_08890W_A), encoding a zinc-finger transcription factor of the C_2_H_2_ family, in a systematic screen of genes whose overexpression contributes to *C. albicans*’ morphological changes. Conditional overexpression of *ORF19.217* with the strong tetracycline-inducible promoter (P*_TET_*) resulted in a hyperfilamentous phenotype. We examined growth of the *orf19.217* knockout-mutant in different hypha-inducing conditions and found that the mutant still formed hyphae under standard hypha-inducing conditions. To further investigate the function of Orf19.217 in *C. albicans*, we combined genome-wide expression (RNA-Seq) and location (ChIP-Seq) analyses. We found that Orf19.217 is involved in regulatory processes comprising hyphal morphogenesis and iron acquisition. Comparative analysis with existing *C. albicans* hyphal transcriptomes indicates that Orf19.217-mediated filamentation is distinct from a true hyphal program. Further, the *orf19.217* knockout-mutant did not show increased sensitivity to iron deprivation, but *ORF19.217* overexpression was able to rescue the growth of a *hap5*-mutant, defective in a subunit of the CCAAT-complex, which is essential for iron acquisition. This suggested that Orf19.217 is involved in regulation of iron acquisition genes during iron deprivation and acts in a parallel pathway to the established CCAAT-complex. Interestingly, the *orf19.217*-mutant turned out to be defective in its ability to form filaments under iron-deficiency. Taken together our findings propose that the transcription factor Orf19.217 stimulates expression of the hyphal regulators *EFG1* and *BRG1* to promote filamentous growth under iron deprivation conditions, allowing the fungus to escape these iron-depleted conditions. The transcription factor therefore appears to be particularly important for adaptation of *C. albicans* to diverse environmental conditions in the human host. In regard to the newly identified functions, we have given the regulator the name Irf1, Iron-dependent Regulator of Filamentation.

## Introduction

*Candida albicans* is a part of the human microbiome and predominantly colonises the gastrointestinal tract of healthy individuals (Nash et al., 2017). However, under certain host conditions, the fungus can switch from a commensal to a pathogenic lifestyle, leading to superficial as well as systemic infections. After *Cryptococcus*, *C. albicans* and related *Candida* species are the main cause of invasive fungal infections (Brown et al., 2012; Bongomin et al., 2017).

*C. albicans* high adaptability is of central importance for survival in the human host and is directly linked to its ability to alter its morphological states. *C. albicans* is optimally adapted to thrive in very diverse environmental conditions and can even survive attacks by the immune system. One of the most important virulence characteristics is the reversible change from a yeast- to hyphal-cell form. This morphological transition allows the fungus to penetrate body tissues or escape from phagocytes (Lorenz et al., 2004; Erwig and Gow, 2016). Hyphae also exhibit enhanced adhesive properties, which are particularly important in the formation of biofilms, a special form of colonisation by a variety of microbes (Kumamoto and Vinces, 2005). Hyphae are also important for reaching new environments, for example to escape nutritional deficiencies (Cullen and Sprague, 2012; Arkowitz and Bassilana, 2019).

To protect itself from invading pathogens, the human body has developed various strategies, including the limitation of essential metals, also known as nutritional immunity (Weinberg, 1975). In this defence mechanism, growth-limiting trace metals, especially iron and zinc, are assimilated immediately after intestinal release and bound in appropriate storage tissues. This rapidly limits circulating trace metals available for uptake and use by pathogens. As a result, pathogens have developed mechanisms to evade the nutritional immunity strategy (Cassat and Skaar, 2013).

In *C. albicans*, three major iron acquisition strategies have been identified: (i) the reductive system, (ii) a siderophore uptake system and (iii) the haemoglobin iron uptake system (Fourie et al., 2018). Approximately two-thirds of the potentially available iron is bound to heme, which is mainly found in the oxygen carrier haemoglobin in the host (Ganz and Nemeth, 2015); accordingly, the fungus has evolved a mechanism to extract heme iron from the host. The uptake relies on the extracellular GPI-anchored heme receptors Rbt5 and Pga7, and the environmentally secreted protein Csa2 (Kornitzer and Roy, 2020). The three proteins belong to the CFEM (Common in Fungal Extracellular Membrane) family of proteins, which is mainly defined by a group of eight cysteines with conserved spacing (Kulkarni et al., 2003). The exact mechanism by which the heme iron is released from the globin is still unclear, but the uptake of the heme iron probably occurs by means of a transfer cascade that starts at Csa2, transfers the heme iron further to Rbt5 in the cell wall and finally transfers it to Pga7, located at the cytoplasmic membrane. The heme iron then enters the cell by endocytosis and is transported by the Myo5/ESCRT system to the vacuole, where the iron is released from the heme (Kornitzer and Roy, 2020). Another mechanism for iron acquisition is the secretion and reuptake of siderophores, molecules with a very high binding affinity for iron (Hider and Kong, 2010). *C. albicans* does not synthesise siderophores itself (Haas, 2003), but produces a siderophore transporter Sit1/Arn1, which is also required for the colonisation of epithelial tissue (Heymann et al., 2002; Hu et al., 2002). Siderophore iron uptake therefore seems to be dependent on siderophores synthetised by other microorganisms. The third strategy used by *C. albicans* to take up iron is the high-affinity or low-affinity reductive systems. In the high-affinity system, ferric iron is first reduced to its ferrous form and transported into the cell by the ferrous transporter complex, where it is re-oxidised by multicopper ferroxidases (Kosman, 2003). A total of 17 potential ferric reductases have been identified in the genome of *C. albicans* (Almeida et al., 2009), two of which have been confirmed experimentally, namely Cfl1/Fre1 and Cfl95/Fre10 (Hammacott et al., 2000; Knight et al., 2002). The iron permease Ftr1 and the multicopper oxidases Fet3 and Ccc2, in particular, are responsible for the transport of ferrous iron into the cell (Eck et al., 1999; Weissman et al., 2002; Ziegler et al., 2011).

On the other hand, high concentrations of trace metals, such as iron or zinc, are toxic both to mammalian and fungal cells. The maintenance of homeostasis is therefore of vital importance for all organisms and the underlying regulatory systems, which include the sensing, acquisition, transport, and storage of trace metals, are correspondingly complex. Maintenance of iron homeostasis in *C. albicans* is mainly regulated by the transcription factors Sef1 and Sfu1, and the heterotrimeric CCAAT-complex (Chen et al., 2011). The GATA transcription factor Sfu1 acts as a repressor of genes responsible for iron acquisition (Lan et al., 2004; Pelletier et al., 2007); in contrast, the Zn_2_Cys_6_ DNA-binding protein Sef1 activates the expression of these genes (Chen et al., 2011). Sef1 also directly activates the expression of *HAP43*, encoding the transcriptionally active subunit of the CCAAT-complex, which is involved in the regulation of genes that play a role in the uptake and in the consumption of iron as well as in the response to oxidative stress (Johnson et al., 2005; Chen et al., 2011; Hsu et al., 2011; Singh et al., 2011). Thus, Sef1 and the CCAAT-complex are positive regulators activated during iron deficiency to stimulate iron uptake, and Sfu1 is an antagonist repressed during iron deficiency that only becomes active at high iron concentrations to inhibit iron uptake, thus counteracting iron-mediated toxicity.

The uncharacterised zinc finger transcription factor Orf19.217 was identified in a systematic screen of genes whose overexpression contributes to morphological changes in *C. albicans* (Chauvel et al., 2012). Interestingly, it was also discovered in mouse infection models that Orf19.217 plays an antagonistic role in mammalian gut colonisation and systemic infection (Perez et al., 2013). In order to better understand the function of Orf19.217 in these important processes, we investigated the regulatory network of Orf19.217 by combining ChIP- and RNA-Seq analyses. We found that Orf19.217 stimulates iron uptake, probably in a pathway parallel to the CCAAT-complex. In addition, Orf19.217 is a positive regulator of pseudohyphal growth directly affecting the expression of known morphogenesis regulators. It has previously been shown that iron deficiency induces the formation of hyphae through the bHLH transcription factor Efg1, the master regulator of morphogenesis in *C. albicans* (Stoldt et al., 1997; Hameed et al., 2008). Our results show that Orf19.217 is required for iron deficiency-induced hyphal formation and identify the transcription factor Brg1 as a downstream effector in this process. We currently assume that the transcription factor Orf19.217 acts at the interface between iron homeostasis and morphogenesis and is therefore of particular importance for adaptation to host-specific conditions. Considering the newly identified functions, we have named this regulator Irf1, for Iron-dependent Regulator of Filamentation.

## Materials and methods

### Cultures and growth conditions

Strains used in this study are listed in **Table S1**. *C. albicans* yeast cells were routinely grown in YPD medium (1% (w/v) yeast extract, 2% (w/v) peptone and 1% (w/v) dextrose) at 30°C. The induction of hyphae was performed on the surface of solid SPIDER medium (1% (w/v) Difco nutrient broth, 2% (w/v) mannitol, 0.2% (w/v) dibasic potassium phosphate) at 37°C. Sensitivity assays were performed in YPD medium at 30°C and 10 mM BPS (bathophenanthrolinedisulfonate, Sigma) was added to the medium to create iron deficient conditions. For iron complemented conditions, an additional 100 μM FeCl_3_ (Sigma) was supplemented to the medium. For this experiment, precultures were grown in YPD medium containing 10 mM BPS. To assess iron deficiency-induced filamentation, 150 μM BPS was added to YPD medium and cultures were incubated at 30°C for 4 to 5 days. For quantification of synthesised riboflavin, cultures were grown in Low Fluorescent medium (LoFlo_glu_, 0.7% (w/v) yeast nitrogen base (without amino acids), 0.25% (w/v) ammonium sulphate, 0.1% (w/v) complete supplement mixture (CSM; MP Biomedicals) and 2% (w/v) glucose) at 30°C. For induction of P*_TET_*, the medium was supplemented with 6 µg/mL anhydrotetracycline (ATc, Sigma). Solid plates were prepared with 2% (w/v) agar.

### Construction of *C. albicans* knockout and overexpression strains

The *irf1* and *hap5* knockout strains were constructed in the SN76 background strain, by combining gene replacement, using *HIS1* and *ARG4* as marker genes as described by Noble and Johnson (2005), with the transient CRISPR\Cas9 system described by Min et al. (2016). After PCR amplification of the replacement cassettes, the *Cas9* encoding sequence and the respective synthetic-guide RNAs (Vyas et al., 2015), the DNA was co-transformed into *C. albicans* cells following a standard LiAc protocol (Sanglard et al., 1996). Successful deletion was confirmed by colony PCR with a set of flanking oligonucleotides and internal oligonucleotides.

For *TET* driven overexpression of *IRF1* the BWP17 derivative strain CEC2907, harbouring the pNIMX plasmid (Chauvel et al., 2012), was transformed with CIp10-derived plasmids from our collection, following gateway cloning of the *IRF1* entry plasmid (Legrand et al., 2018). As a control, the empty CIp10 vector was integrated. The transformation was performed using a standard LiAc protocol (Sanglard et al., 1996). Successful integration of the plasmids into the *RPS1* locus was confirmed by colony-PCR (Chauvel et al., 2012). Likewise, the *irf1* and *hap5* mutant strains were transformed with these plasmids.

Knockout strains from the Homann collection (Homann et al., 2009), comprising TF022 (ΔΔ*brg1*), TF115 (ΔΔ*tec1*), TF132 (ΔΔ*ahr1*) and TF156 (ΔΔ*efg1*) were transformed with either pNIM1 (Park and Morschhauser, 2005) or a pNIM1-derived *IRF1* overexpression plasmid (Cabral et al., 2014). Oligonucleotides used in this study are listed in **Table S1**.

### Microscopy

Aliquots of liquid cultures were washed twice with PBS, cells were transferred to microscope slides and examined using a Leica DM RXA microscope (Leica Microsystems) with an oil-immersed 100x objective (1.4 N/A). For chitin staining, cells were incubated with 2 µg/mL of calcofluor white for 5 min and washed twice with PBS prior to observation. Fluorescent signal was collected with the single band pass filter A (Leica), and images were captured with a Hamamatsu ORCA II-ER cooled CCD camera. Macro photographs of individual colonies were taken from the bottom of the Petri dish using a 40x lens. Images were processed with ImageJ.

### Quantification of riboflavin

Riboflavin secretion was quantified in the *irf1* knockout strain and the *IRF1* overexpression strain. Overnight cultures were used to inoculate 5 mL fresh LoFlo_glu_ medium, supplemented with 6 µg/mL anhydrotetracycline, to an OD_600_ of 0.1 and grown at 30°C for 72 h. The OD_600_ of the stationary phase cultures were measured and the supernatants were cleared by centrifugation. Two hundred µL of the cleared supernatants were transferred to a microtiter black transparent 96 well plate and fluorescence emission between 420 and 720 nm was recorded immediately after excitation at 450 nm in an Infinite M Flex microtiter plate reader (Tecan). As a reference the spectrum of a 0.5 mg/L riboflavin solution in LoFlo_glu_ medium was measured.

### Chromatin immunoprecipitation followed by high-throughput sequencing

Strains expressing either Irf1 with a C-terminus hemagglutinin (HA) tag (CEC5401, P*_TET_*-*IRF1*-HA) or the unmodified protein (CEC5387) from the *TET*-promoter were grown overnight in YPD medium at 30°C and then used to inoculate 50 mL of fresh YPD medium to an OD_600_ of 0.2. *TET*-promoter induction was achieved by adding 6 µg/mL ATc to the medium, and strains were allowed to grow for 4 h at 30°C. The experiment was performed in triplicate and all subsequent steps were completed as described by Znaidi et al. (2018), comprising DNA-cross-linking, chromatin shearing and immunoprecipitation (ChIP). Integrity of the precipitated DNA was confirmed by Fragment Analyzer (Agilent) and following quantification using the Qubit dsDNA HS assay kit (Agilent), 10 ng were used for DNA library preparation with the TruSeq ChIP library preparation kit (Illumina). Size selection of the corresponding library was performed by two consecutive AMPure XP bead (Beckman) steps. Sequencing was performed on a NextSeq 500 system (Illumina), and reads were mapped on the available *C. albicans* genome sequence assembly 22 (candiagenome.org) using Hisat2 (Kim et al., 2019). Transcription factor binding sites were identified using bPeaks (Merhej et al., 2014) and visually inspected with IGV (Thorvaldsdottir et al., 2013). The Irf1 binding dataset is available in **Table S2**.

### Transcriptome analyses

Genome-wide transcript profiles were obtained on three biological replicates (i) for the *irf1* knockout strain (CEC6056) using the corresponding parental strain (CEC4664) as a reference and (ii) for strains overexpressing *IRF1* from the strong *TET*-promoter (P*_TET_*-*IRF1*) (CEC5387) after 2 h and 4 h of induction using the control strain harbouring the empty vector (CIp10) (CEC5348) as a reference. All strains were grown overnight in YPD medium and diluted in fresh YPD medium to OD_600_ of 0.1 and grown for 4 h at 30°C. For *TET*-mediated overexpression the medium was supplemented with 6 µg/mL ATc, and an aliquot of the cells was taken after 2 h of growth. Cells were mechanically disrupted by bead beating with glass beads and total RNA was isolated using the RNA extraction kit from Qiagen. Libraries were prepared with the TruSeq stranded mRNA library kit (Illumina) followed by sequencing on a Hiseq2500 system (Illumina). After quality control assessment and adapter trimming, corresponding reads were mapped on the assembly 22 C*. albicans* genome sequence (candiagenome.org) using Hisat2 (Kim et al., 2019). DEseq2 (Love et al., 2014) was used for differential expression analysis assuming a fold-change of 1.5 with P-adj< 0.05 as significant. The Irf1 transcript profiles are available in **Table S2**.

### Quantitative real time PCR analyses

The *irf1* knockout strain (CEC6056), the *IRF1* overexpression strain (CEC5387) and a corresponding control strain (CEC5348) were grown overnight in YPD medium and used to inoculate 16 mL of YPD medium or YPD medium supplemented with 150 μM BPS. To induce the *TET*-promoter, an additional 6 µg/mL of ATc was added to the medium. The cultures were grown for 4 h at 30°C and then total RNA was extracted using the RNA extraction kit from Qiagen. Reverse transcription was performed for 2 μg total RNA with the QuantiTect Reverse Transcription kit (Qiagen). The qPCR was performed in MicroAmp Optical 96-Well Reaction Plates (Applied Biosystems) using the MX3000 Real-Time PCR System and software (Stratagene) with the 2X Takyon Rox SYBR MasterMix dTTP blue (Eurogentec) following the manufacturer instructions. Relative transcript levels of *IRF1*, *CFL4*, *CFL5*, *FTR1*, *EFG1*, *AHR1*, *TEC1* and *BRG1* were determined following the ΔC_T_ method using *ACT1* as a calibrator.

The corresponding oligonucleotide sequences are available in **Table S1**.

### Transcription factor binding motif prediction

The Irf1-HA peak regions identified by ChIP-Seq were analysed using MEME-ChIP (Ma et al., 2014) with standard parameters, to determine motif enrichment. Known transcription factor binding motifs from YEASTRACT+ (Monteiro et al., 2020) were used as references for motif comparison.

### Gene ontology analyses

The functional classification of identified target genes was performed by gene ontology analysis, using FungiFun2 (https://elbe.hki-jena.de/fungifun/; Priebe et al. (2015)).

### Sequence alignment analysis

Amino acid sequences for *C. albicans* Irf1 and *Saccharomyces cerevisiae* Cmr3 were obtained from CGD (candidagenome.org) and SGD (yeastgenome.org), respectively. Clustal omega version 1.2.4 (Goujon et al., 2010) was used to generate the sequence alignment.

### Data availability

Data derived from ChIP-Seq and RNA-Seq is available at omnibus under the numbers GSE207033 and GSE207073, respectively.

## Results

### *IRF1* overexpression induces a hyperfilamentous phenotype and the secretion of riboflavin

The ability of *C. albicans* to switch between the yeast and hyphal cell forms is considered an intrinsic virulence feature of the fungus (Jacobsen et al., 2012). In order to identify new regulators of morphogenesis, a screen for genes whose overexpression leads to a change in the cell shape of *C. albicans* through overexpression was performed and identified *IRF1*, encoding a so far uncharacterised C_2_H_2_ zinc finger transcription factor (Chauvel et al., 2012). Overexpression of *IRF1* with either the *PCK1-* or the *TET*-promoter was able to induce hyphal growth under conditions that normally favour the appearance of the yeast cell form (Chauvel et al., 2012).

**Figure 1:**
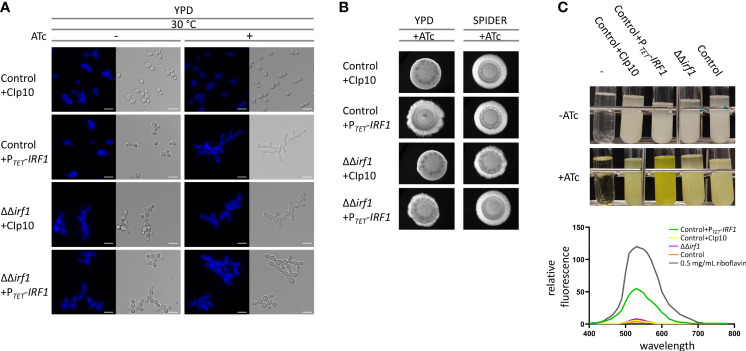
Overexpression of *IRF1* induces filamentation and the production of riboflavin. **(A)** *C. albicans* control strain harbouring either the empty CIp10 plasmid or the P*_TET_*-*IRF1* plasmid and the *irf1* knockout strain harbouring either the control CIp10 plasmid or the P*_TET_*-*IRF1* plasmid were grown in YPD medium at 30°C to logarithmic phase either in the presence or absence of anhydrotetracycline (ATc). Calcofluor-white was used for staining chitin in the cell wall. Scale bars = 3 μm. **(B)** Colony morphology of indicated *C*. *albicans* strains grown on YPD or SPIDER medium supplemented with anhydrotetracycline (ATc). Microphotographs were taken after 3 days of incubation at 37°C. **(C)** *C. albicans* control strain harbouring either the empty CIp10 plasmid or the P*_TET_*-*IRF1* plasmid and the *irf1* knockout strain and its parental strain (control) were grown in LoFlo_glu_ medium supplemented or not with anhydrotetracycline. Top: culture after 72h growth; -: blank. Bottom: supernatants of indicated strains were cleared and the fluorescence spectra were measured upon excitation at 450 nm. Data are expressed as fluorescence relative to OD_600_ of the cultures as average and SEM. As a control the spectrum of 0.5 mg/mL riboflavin was measured.

In addition to our previous results, which were obtained exclusively by conditional overexpression, we investigated the phenotype of the *irf1*-knockout mutant in hypha-inducing condition. For this purpose, a knockout for *IRF1* was generated in one step using a transient CRISPR\Cas9 system (Min et al., 2016) in the SN76 background (Noble and Johnson, 2005), yielding CEC6054. The complementation of the mutant was accomplished by integrating a tetracycline-inducible *IRF1* overexpression plasmid at the *RPS1* locus (CEC6058); the empty vector (CIp10) was integrated as a control (CEC6056). For morphological investigation, strains were grown in yeast-inducing conditions, i.e., YPD medium at 30°C for 4 hours, in the absence or presence of anhydrotetracycline (ATc), which is used to induce the *TET*-promoter, and then examined by microscopy. Accordingly, no morphological abnormalities were observed in the control strain upon addition of ATc (**Figure 1A**, Control+CIp10). As previously shown (Chauvel et al., 2012), overexpression of *IRF1* induced the formation of filamentous growth in the control strain. The elongated cell shape and the distinct constrictions at the septal sites are indicative of pseudohyphae. In contrast, the *irf1*-mutant formed long cell chains, indicating a change in cell surface properties. As the cell chains could be resolved by gentle vortexing, a cell separation defect can therefore be excluded. The morphology of the cells essentially corresponded to that of yeast cells. Similar to the overexpression in the control strain, the overexpression of *IRF1* in the mutant induced the formation of pseudohyphae (**Figure 1A**).

Next, we looked at hyphal inducing conditions by examining the morphology of the colonies. Unexpectedly, the *irf1*-mutant showed no hyphae defect on several media conducive to hyphal growth, namely SPIDER medium (**Figure 1B**), Lee’s medium, Serum-containing medium or RPMI (data not shown). The colony morphology of the overexpression strains resembled that of the control strain (**Figure 1B**).

Although our results confirm that overexpression of *IRF1* induces a hyperfilamentous phenotype, since the mutant does not exhibit a hyphae defect, it is more likely that Irf1 may play a minor or condition-dependent role in the regulation of (pseudo)hyphal growth of *C. albicans*.

After a longer incubation period in YPD medium, we surprisingly observed a strong yellow-green colouring of the culture medium when *IRF1* was overexpressed (**Figure 1C**). Fluorescence emission analysis of the cell-free culture supernatant revealed that overexpression of *IRF1* leads to the secretion of riboflavin (vitamin B2) (**Figure 1C** lower panel). In the mutant, no difference in riboflavin secretion could be detected under these conditions. In contrast to humans, *C. albicans* is able to synthesise this essential vitamin itself, which makes it an interesting target for the development of new antifungal agents (Abbas and Sibirny, 2011; Dietl et al., 2018). Riboflavin acts as a precursor molecule in the synthesis of flavin mononucleotides (FMN) and flavin adenine dinucleotides (FAD), which are required for the synthesis of flavoproteins (Mewies et al., 1998). Flavoproteins are involved in numerous metabolic processes, including respiratory chain energy production and fatty acid oxidation (Gnandt et al., 2016; Dietl et al., 2018). Recently, it has been shown that iron deficiency stimulates the production and secretion of riboflavin in *C. albicans* (Demuyser et al., 2020). The increased secretion of riboflavin upon overexpression of *IRF1* thus suggests that Irf1 is either directly involved in the regulation of genes important for riboflavin biosynthesis or indirectly, for instance through the regulation of iron homeostasis.

### The Irf1 gene regulatory network

**Figure 2:**
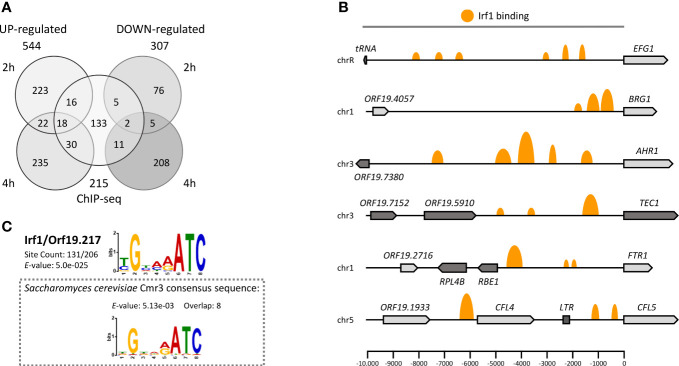
The Irf1 regulatory network in *C*. *albicans*. **(A)** Venn diagram showing the number of significantly up- and downregulated genes identified by RNA-Seq after 2 or 4 hours of overexpression of *IRF1* from a tetracycline inducible promoter in YPD medium and the number of direct target genes identified by ChIP-Seq under the same conditions. **(B)** Schematic overview of Irf1 binding sites within selected promoter regions of identified target genes. The size of the orange marks reflects the log fold change between number of reads in the IP and control samples. **(C)** The binding motif of Irf1 was determined using the ChIP-MEME suite (https://meme-suite.org/). The identified motif is very similar to the binding motif of Cmr3 from *S. cerevisiae*.

To comprehensively define the regulatory network of Irf1 and better understand how *IRF1* contributes to the regulation of *C. albicans* morphogenesis, a transcript profile of the whole genome of the strain overexpressing *IRF1* with the *TET*-promoter was established. As a reference, we used the parental strain carrying the empty vector. Both strains were cultured in ATc-containing YPD medium at 30°C and the transcriptome was determined by RNA-Seq after 2 h (early time point) and 4 h (later time point) overexpression. Transcriptional changes included 279 upregulated and 88 downregulated genes for the earlier time point and 305 upregulated and 226 downregulated genes for the later time point (fold-change ≥1.5 or ≤ -1.5, P < 0.05). Surprisingly, only moderate concordance of modulated genes was detected between the early and late time points of *IRF1* overexpression (**Figure 2A**).

In addition, to distinguish between Irf1 direct and indirect target genes, genome-wide binding of Irf1 was investigated by chromatin immunoprecipitation followed by next generation sequencing (ChIP-Seq) in strains expressing either HA-tagged or unmodified *IRF1*, both under the control of the *TET*-promoter in ATc-containing YPD medium for 4 hours at 30°C. Using the bPeaks program (Merhej et al., 2014), we identified 242 binding sites of Irf1 localised mainly upstream of ORFs on all 8 chromosomes (**Table S2**). Overall, the 242 binding sites matched 215 target genes, assuming that two ORFs sharing the same bound promoter are both direct targets of Irf1 (**Figure 2A**). Many promoter regions had more than one binding site of Irf1; for instance, 6 binding sites were identified for the 10-kb promoter region of *EFG1* (**Figure 2B**). In conclusion, of the 215 direct target genes identified, 64 genes showed stimulation of their expression mediated by *IRF1* overexpression and 18 genes were inhibited, indicating that Irf1 acts as both an activator and a repressor of gene expression.

The identified binding sequences of Irf1 were used for motif enrichment analysis with the program MEME-ChIP (Ma et al., 2014). The analysis revealed that Irf1 binding regions are enriched in the sequence (T/C)GNN(A/G)ATC. Comparison with the *S. cerevisiae* binding motif database (Yeastract) revealed that the identified binding motif is identical to that of Cmr3 (*YPR013C*), the Irf1 closest homolog in *S. cerevisiae*. However, protein sequence analysis revealed that the two proteins share only 19.2% identity, which is mainly conserved in the zinc finger coding sequences (**Figure S1**).

### The *IRF1* transcript profile is indicative of filamentation but not of true hyphae

**Figure 3:**
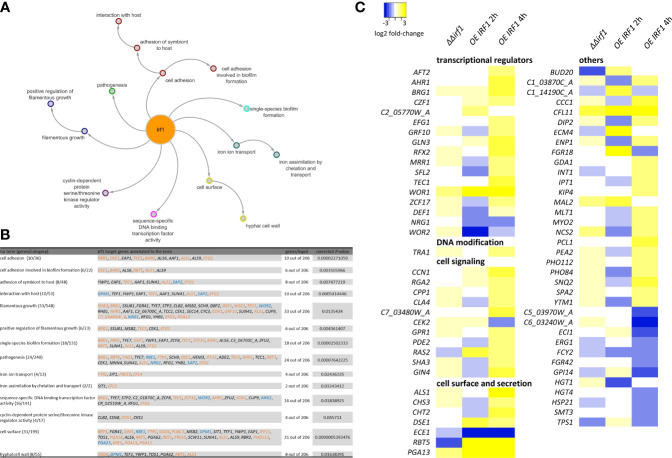
Functional analysis of Irf1 target-genes. Gene-ontology analysis was used to functionally classify identified target genes. The corresponding GO categories are presented **(A)** as a network with corresponding subcategories and **(B)** summarised in a table. For this analysis, only genes that could be identified by ChIP-Seq (direct target-genes) were considered. Genes whose names are highlighted also show a significant difference in expression when *IRF1* is overexpressed (blue = down, orange = up, black = no significant change). **(C)** Heatmap of expression differences (log2 fold-changes) of identified genes assigned to the GO category filamentous growth. The analysis also included genes that did not have a direct binding site of Irf1, but whose expression was altered by the overexpression of *IRF1* or by deletion, in the *irf1* mutant transcriptome.

To obtain an overview of the pathways/processes modulated by Irf1, gene ontology (GO) analysis was performed, considering only genes classified as direct target genes of Irf1 (obtained by ChIP-Seq). Identified target genes of Irf1 encode proteins that play a role in cell adhesion (e.g. *ALS1*, *ALS6*, *ALS9*, *AAF1*), cell surface (e.g. *RBT1*, *RBT4*, *RBT5*, *YWP1*, *PGA11*, *PGA13*, *PGA18*), interaction with the host (e.g. *ALS1*, *SAP2*, *GPM1*, *EAP1*), filamentous growth (e.g. *CEK1*, *MSB2*, *EFG1*), pathogenesis, and, interestingly, iron ion transport (e.g. *FTR1*, *SIT1*, *FRE10*, *CFL4*) and iron assimilation by chelation and transport (*SIT1* and *CFL5*). In addition, a large number of genes encoding transcription factors (16 out of 206 target genes) were identified, including positive regulators of hyphal morphogenesis such as *BRG1*, *TEC1*, *AHR1* and *EFG1*, but also negative regulators such as *TCC1*, *RFG1* and *NRG1* (**Figures 3A, B**).

In summary, the results suggested that Irf1 stimulates the expression of positive regulators of hyphal morphogenesis and inhibits negative regulators, which might explain the hyperfilamentous phenotype when *IRF1* is overexpressed. The functions of the identified Irf1 target genes are centred on hyphal formation, the modulation of the fungal cell surface and additionally on the acquisition of iron.

To gain a better understanding of the underlying mechanisms of the morphological change upon overexpression of *IRF1* at the gene regulatory level, we constructed a heat map based on all genes that showed a change in their expression at early (2 h) or late (4 h) time points in the transcriptome analysis and could be assigned to the GO category filamentous growth (**Figure 3C**). We also included the transcriptome analysis of the *irf1*-mutant in this analysis (**Table S2**). Most of the identified transcriptionally modulated genes encode transcription factors themselves, proteins involved in signal transduction and proteins localised in the cell wall or secreted peptides. The transcriptional changes were most pronounced after 4 h overexpression of *IRF1*, whereas the *irf1*-mutant showed almost no significant changes. Apart from *RBT5* and *ECE1*, no transcriptional change could be detected in any of the conventional hyphal indicator genes (Martin et al., 2013). *ECE1* encodes the candidalysin, which is secreted by hyphal cells and is important in *Candida*-host interactions (Moyes et al., 2016). Surprisingly, the expression of *ECE1* was repressed as a consequence of *IRF1* overexpression, whereas hyphal stimulating conditions activate the expression of *ECE1* (Birse et al., 1993), suggesting that an additional hyphal-stimulating signal is missing. The transcriptome analyses were performed in YPD medium at a temperature of 30°C, a growth condition that favours the formation of yeast cells. However, the transcriptome data indicate that overexpression of *IRF1* induces filamentous growth of the fungus, but in agreement with our phenotype data (**Figure 1A**), only induces pseudohyphal growth, not the formation of true hyphae. Among the transcriptionally regulated genes were components of the Cek MAP kinase pathway (*CPP1*, *CEK2*), and components of the Ras/cAMP/PKA pathway (*GPR1*, *RAS2*, *PDE2*), both involved in the regulation of morphogenesis (Basso et al., 2019), and components of the pathway controlling cell polarity (*CCN1*, *RGA2*).

A combination of different signalling pathways therefore seems to be involved in the establishment of the hyperfilamentous phenotype of *IRF1*.

### Irf1 target genes are involved in iron acquisition

**Figure 4:**
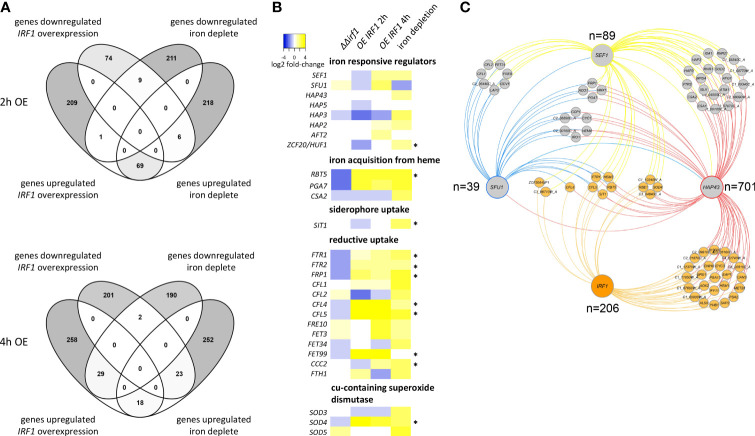
Comparative analysis of the *IRF1* transcriptome with the iron deficiency transcriptome. **(A)** Transcriptome data of *C*. *albicans* cells incubated under iron deficiency were taken from Lan et al. (2004) and compared with the transcriptome data of *C. albicans* overexpressing *IRF1* after 2 hours (upper panel) and after 4 hours (lower panel). **(B)** Heatmap of expression differences (log2 fold-changes) of genes involved in iron homeostasis. (* indicates that the gene is a direct target of Irf1). **(C)** Interaction network of transcriptional regulators involved in maintaining iron homeostasis. The corresponding target genes of the respective transcription factors were obtained from pathoyeastract (http://pathoyeastract.org/) and graphically displayed with Cytoscape (Shannon et al., 2003).

GO analysis had revealed that Irf1 is also involved in the regulation of genes involved in iron acquisition. In addition, we had observed that long-term overexpression of *IRF1* contributes to the production and secretion of riboflavin (**Figure 1C**), which could be related to an impairment of iron homeostasis (Demuyser et al., 2020). Therefore, we compared our transcriptome data with the published transcript profile of cells cultured under iron deprivation (Lan et al., 2004). The growth conditions for transcriptome analysis were similar to ours, the wild-type strain was grown in YPD medium at 30°C and treated with the iron chelator BPS (bathophenanthrolinedisulfonate) to induce iron deficiency conditions (Lan et al., 2004). The comparative transcript analysis revealed that at the early time point of *IRF1* overexpression (2 hours), a total of 85 genes matched the genes regulated under iron deficiency, with the expression of 69 genes being equivalently activated under both conditions, and 9 genes being equivalently repressed (**Figure 4A**). At the later time point of *IRF1* overexpression (4 hours), 72 genes coincided, but in contrast to the early time point, some genes showed opposite regulation, 29 genes activated by *IRF1* were repressed under iron deficiency, and 23 genes inhibited by *IRF1* were activated under iron deficiency (**Figure 4A**), only 18 genes were equivalently activated, and 2 genes were repressed.

The results clearly showed that Irf1 is involved in the regulation of some of the genes regulated by iron deprivation, but the transcriptional programme seems to be more in line with the iron deprivation profile at the early time point. At the later time point, a compensatory response has probably already occurred.

To investigate the role of Irf1 in the regulation of iron homeostasis, we specifically examined the expression of genes that are either themselves involved in the regulation of iron homeostasis or encode components of iron acquisition (**Figure 4B**). The expression pattern of genes encoding iron regulators shows that at the early time point of *IRF1* overexpression, both the positive iron regulators *SEF1* and subunits of the CCAAT-complex, *HAP5* and *HAP3*, and the negative regulator *SFU1* are inhibited. However, expression is derepressed at the later time point and, conversely, expression of both *SEF1* and *SFU1*, which play antagonistic roles in the regulation of iron homeostasis (Chen et al., 2011), is induced.

Both the pathway for iron acquisition by heme (*RBT5*, *PGA7*) and components of the reductive pathway (*FTR1*, *FTR2*, *CFL4*, *CFL5*, *FRE10*, *FET3* and *FET99*) exhibited strong activation upon overexpression of *IRF1* at both time points (**Figure 4B**). Similarly, these genes exhibited strongly increased expression under iron deficiency conditions. In contrast, in the *irf1*-mutant, the transcripts of these genes were repressed, suggesting that they are under the direct control of Irf1. Consistent with this, we identified binding sites of Irf1 in the regulatory sequences of some of these genes, including *RBT5*, *SIT1*, *FTR1*, *FTR2*, *FRP1*, *CFL4*, *CFL5*, *FET99*, *CCC2* and *SOD4* (as indicated by *, **Figure 4B**). We were thus able to identify Irf1 unambiguously as a regulator of genes directly involved in iron acquisition. Our transcriptome data do not indicate a direct influence of Irf1 on riboflavin biogenesis (**Figure S2**), although we could detect direct binding of Irf1 in the regulatory sequence of the *RIB3* gene, which encodes an enzyme of the riboflavin synthesis (Echt et al., 2004). It should also be noted that Irf1 is a good example of the utility of the overexpression strategy in elucidating important processes in *C. albicans* (Rai et al., 2022), as the mutant study did not reveal any insight into the function of the protein.

### The iron co-regulatory network

Next, we wanted to uncover the position of Irf1 in regulating the maintenance of iron homeostasis. To this aim we compared the gene regulatory network of Irf1 with the target genes of Sef1, Sfu1 and Hap43, the three main regulators of iron acquisition and homeostasis (Chen et al., 2011). The respective associations were obtained from PathoYeastract (Monteiro et al., 2020) and used to construct the iron co-regulatory network (**Figure 4C** and **Figure S3**). Reciprocal regulation of transcription factors could only be detected between *SFU1*, *SEF1* and *HAP43*; *IRF1* could not be identified as a target gene of these regulators (**Figure 4C**). We only detected a direct association of Irf1 and Sef1 with *ZCF20*, which encodes a transcription factor involved in the regulation of iron homeostasis orthologous to *S. cerevisiae HAP1* (Pfeifer et al., 1989). Irf1 therefore does not appear to be located upstream of the other iron homeostasis regulators. Target genes regulated by all four transcription factors include *SIT1*, encoding a ferrichrome siderophore transporter (Heymann et al., 2002), *CFL5*, a ferric reductase (Knight et al., 2005), *FTR1*, encoding a high-affinity iron permease (Ramanan and Wang, 2000), the cell wall protein-encoding gene *RBT5*, which is involved in iron acquisition from heme (Kuznets et al., 2014) and *HEM3*, involved in heme biosynthesis (Kurtz and Marrinan, 1989). Other common target genes comprise *CFL4*, *SOD4*, *ZCF20*, *RBE1* and 3 uncharacterized ORFs (**Figure 4C**). The evaluation of the network suggests that Irf1 acts in parallel with the established signalling pathways.

### Irf1 is involved in regulation of genes relevant for commensalism of *C. albicans*

Based on the previously suggested antagonistic function of Irf1 in mammalian gut colonisation and systemic infections (Perez et al., 2013), we also compared our *IRF1* transcriptome data with the transcript profile of *C. albicans* cells commensally living in the GI tract of mice (Rosenbach et al., 2010), with 99 genes showing similar expression changes under both conditions (**Figure S4A**). The detailed comparison with the Irf1-regulon revealed that the expression of 36 genes directly modulated by the transcription factor were involved in processes such as iron transport (*FTR1*), haemoglobin utilisation (*RBT5*), riboflavin biosynthesis (*RIB3*), filamentous growth (*AHR1*, *ALS1*, *CUP9*, *RBT5*, *SHA3*, *STP2*, *SUN41*, *TEC1*, *YHB1*) and cell adhesion (*AHR1*, *ALS1*, *RBT5*, *TEC1*) (**Figure S4B**). Consequently, these processes seem to be very fundamental for the commensal life of *C. albicans* in the GI tract, which also supports a role of the Irf1 transcription factor in controlling a gene expression program specifically active in the host.

### Irf1 mediates increased resistance to iron chelation, independent of the CCAAT-complex

**Figure 5:**
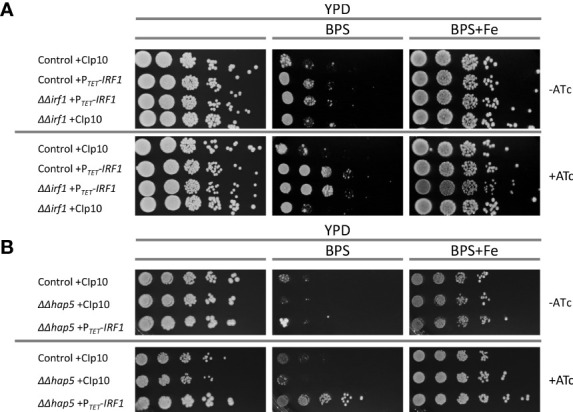
Function of Irf1 in iron deficiency conditions. Spot dilution assays were performed on YPD medium, on YPD medium supplemented with the iron-chelator BPS and YPD medium supplemented with BPS (10 mM) but also iron excess (100 μM FeCl_3_). For the induction of the TET-promoter, anhydrotetracycline (+ATc) was also added to the medium when indicated. **(A)** *C. albicans* control strain and the *irf1* mutant strain harbouring either the empty plasmid CIp10 or the overexpression plasmid P*_TET_*-*IRF1* were grown over night in YPD medium supplemented with anhydrotetracycline and then gradually diluted and pipetted onto the media. Macrophotographs were taken after 2 days of incubation at 30°C. **(B)** Similarly, growth of *hap5* mutant strains harbouring either the empty plasmid CIp10 or the overexpression plasmid P*_TET_*-*IRF1* were investigated by spot dilution assay under the same conditions.

Based on our transcriptome data and the analysis of the gene regulatory network of Irf1, we assumed that Irf1 stimulates the acquisition of iron. The transcription factor could therefore be important for *C. albicans* in adapting to iron deficiency conditions. To test our hypothesis, the sensitivity of *IRF1* overexpression strains and the *irf1*-mutant to the iron chelator BPS was investigated. As expected, *IRF1* overexpression was able to rescue *C. albicans* grown under iron deficiency conditions (**Figure 5A**, BPS, +ATc). In contrast, the mutant exhibited the same growth behaviour as the control strain but was also rescued by overexpression of *IRF1* (**Figure 5A**). Addition of supplementary FeCl_3_ abolished the growth defect induced by BPS. Since the mutant does not show increased sensitivity, it can be assumed that Irf1 is functionally redundant with respect to its function in the regulation of iron acquisition genes.

Analysis of the iron co-regulatory network suggested that Irf1 acts in parallel with the signalling pathway mediated by Sef1 and the CCAAT-complex. Accordingly, we tested whether overexpression of *IRF1* is able to compensate for the growth defect of the *hap5*-mutant; *HAP5* encodes a subunit of the CCAAT-complex and it was shown that the *hap5*-mutant, like the *hap43*-mutant, is extremely sensitive to the iron chelator BPS (Johnson et al., 2005; Homann et al., 2009). Strikingly, overexpression of *IRF1* caused a marked resistance to BPS in the *hap5*-mutant strain (**Figure 5B**). Therefore, *IRF1* overexpression can bypass the CCAAT-complex to activate expression of iron acquisition genes. This result therefore supports our assumption that Irf1 either functions in parallel with the CCAAT-complex-mediated signalling pathway or is located further downstream of it.

### Iron acquisition and filamentous growth are co-regulated

**Figure 6:**
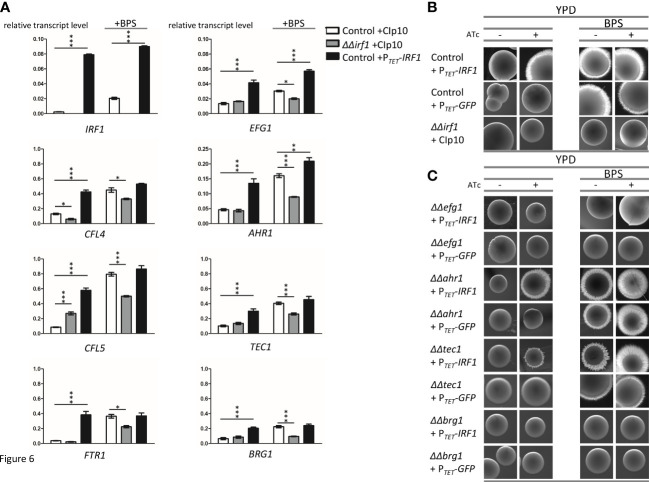
Irf1 is dispensable for the regulation of genes involved in iron uptake but critical for iron deficiency-induced filamentation. **(A)** qRT-PCR was used to investigate the influence of Irf1 on the expression of genes that play a role in iron uptake (*CFL4*, *CFL5*, *FTR1*, left side) or that are important for filamentation (*EFG1*, *AHR1*, *TEC1* and *BRG1*, right side). A control strain harbouring either the empty plasmid CIp10 (white bars) or the overexpression plasmid P*_TET_*-*IRF1* (black bars) and the *irf1* mutant strain harbouring the CIp10 plasmid (grey bars) were grown to logarithmic phase in YPD medium or YPD medium supplemented with BPS (150 μM) and used for RNA extraction and subsequent cDNA synthesis. Data are expressed as average with SEM. Asterisks indicate significant differences in gene expression levels compared to those of the control strain determined by a two-tailed Student’s *t*-test. (*) *P*< 0.05; (**) *P* < 0.01; (***) *P* < 0.001. **(B)** A low concentration of BPS (150 μM) was used to activate filamentation induced by iron starvation of *C. albicans* control strain harbouring the pNIM1 derived P*_TET_*-*IRF1* overexpression plasmid and the *irf1* mutant strain harbouring the empty control plasmid grown on the surface of YPD plates either supplemented with anhydrotetracycline (+ATc) or without. Macrophotographs of single colonies were taken after 4-5 days of incubation of the plates at 30°C. **(C)** Similarly, mutant strains of known hyphal regulators harbouring the pNIM1 derived P*_TET_*-*IRF1* overexpression plasmid were investigated for their ability to contribute to iron starvation induced filamentation as compared to the WT + P*_TET_*-*IRF1* control (see top panels of **Figure 6B**).

To confirm the Irf1-dependent regulation of genes under iron deficiency conditions, their transcript levels were determined in the *irf1*-mutant and the *IRF1* overexpression strain, in YPD medium or in YPD medium supplemented with BPS. While the transcript level of *IRF1* itself was increased more than 40-fold by *TET*-promoter induced overexpression (**Figure 6A**, left panel), iron deficiency resulted in a 16-fold increased transcript level, indicating that expression of *IRF1* is modulated by iron. The expression of the identified Irf1 target genes *CFL4*, *CFL5* and *FTR1* (**Figure 2B**), which are involved in the acquisition of iron by the reductive pathway, were also upregulated by iron deficiency and by the overexpression of *IRF1*, which agrees with our RNA-Seq data and the published data of the iron deficiency transcriptome (Lan et al., 2004). In the *irf1*-mutant, transcript levels did not reach control strain levels under iron deprivation, but transcripts were still elevated as compared to untreated growth conditions, indicating that other regulators, in the absence of Irf1 function, are contributing to transcriptional induction of those genes under iron deprivation (**Figures 6A** and **S5**). The results thus support the hypothesis that Irf1 plays only a minor role in the stress response to iron deficiency, at least with respect to the regulation of iron acquisition.

Returning to our original hypothesis that Irf1 is a regulator of hyphal morphogenesis, we also examined the expression of identified Irf1 target genes encoding known regulators of hyphal formation (**Figure 2B**). It was previously shown that hypha-inducing conditions also triggered expression changes of the iron acquisition pathways (Luo et al., 2021), indicating that these transcriptional programs are functionally linked. In agreement with our RNA-Seq results, overexpression of *IRF1* under normal growth conditions was able to stimulate the expression of *EFG1*, *AHR1*, *TEC1* and *BRG1*, whereas no difference was observed in the *irf1*-mutant as compared to the control strain (**Figure 6A**, right panel). Interestingly, iron deficiency also caused an induction of the expression of the four transcription factors. Unlike the iron acquisition genes, *EFG1*, *AHR1*, *TEC1* and *BRG1* were not significantly increased in the *irf1*-mutant, but *AHR1* and *TEC1* showed significant increased levels as compared to untreated conditions, suggesting control by other factors (**Figures 6A** and **S5**). Accordingly, Irf1 seems to have a particular function in the modulation of some hyphal regulators under iron deficiency conditions.

### Iron deficiency-induced filamentation relies on Irf1

A functional connection between iron deficiency and hyphal formation could be that the fungus can escape trace element-depleted conditions through targeted hyphal growth by scavenging iron. It has already been shown that iron deficiency in *C. albicans* leads to the induction of hyphal growth, both in liquid and solid media (Hameed et al., 2008). To investigate a possible involvement of Irf1 in this process, colony morphologies of *IRF1* overexpression strain and *irf1*-mutant were examined under iron deficiency conditions (YPD + BPS). As previously observed, overexpression of *IRF1* induced the formation of hyphae on YPD medium as indicated by the fuzzy colony edges (**Figure 6B**). As expected, iron deficiency caused by BPS was able to induce hyphal growth in the control strain, and overexpression of *IRF1* also resulted in pronounced hyphal growth under these conditions (**Figure 6B**). Notably, in contrast, the *irf1*-mutant was unable to filament under iron deficiency (**Figure 6B**), suggesting that Irf1 is an important regulator of the iron deficiency-induced hyphal growth.

To clarify whether *EFG1*, *AHR1*, *TEC1* or *BRG1* are also involved in the regulation of hyphal growth induced by iron deficiency, the corresponding mutant strains were transformed with the *TET-IRF1* overexpression plasmid, and their colony phenotypes were examined under iron deficiency. It has already been shown that *EFG1* is required for hyphal formation induced by iron deficiency (Hameed et al., 2008). Consistently, the *efg1*-mutant failed to form filaments under iron deficiency conditions, as indicated by the smooth colony edges (**Figure 6C**). Interestingly, *IRF1* overexpression in the *efg1*-mutant was also unable to induce hyphal growth, neither under normal growth conditions nor under iron deficiency (**Figure 6C**). Similarly, in the *brg1*-mutant, neither iron deficiency nor *IRF1* overexpression was able to induce hyphal growth (**Figure 6C**). In contrast, both the *ahr1*-mutant and the *tec1*-mutant were able to filament under iron deficiency conditions, although the *tec1*-mutant had slightly fewer filaments. In addition, overexpression of *IRF1* was also able to induce hyphal growth in the mutants under both growth conditions (**Figure 6C**), suggesting that Ahr1 and Tec1 are dispensable for the hyphal program induced by iron deficiency and are also not required for the hyphal programme induced by Irf1, thereby indicating that both programmes are similar. *EFG1* and *BRG1* were both identified as downstream target transcription factors of Irf1, which are required for the expression of the iron deficiency hyphal programme mediated by Irf1.

## Discussion

**Figure 7:**
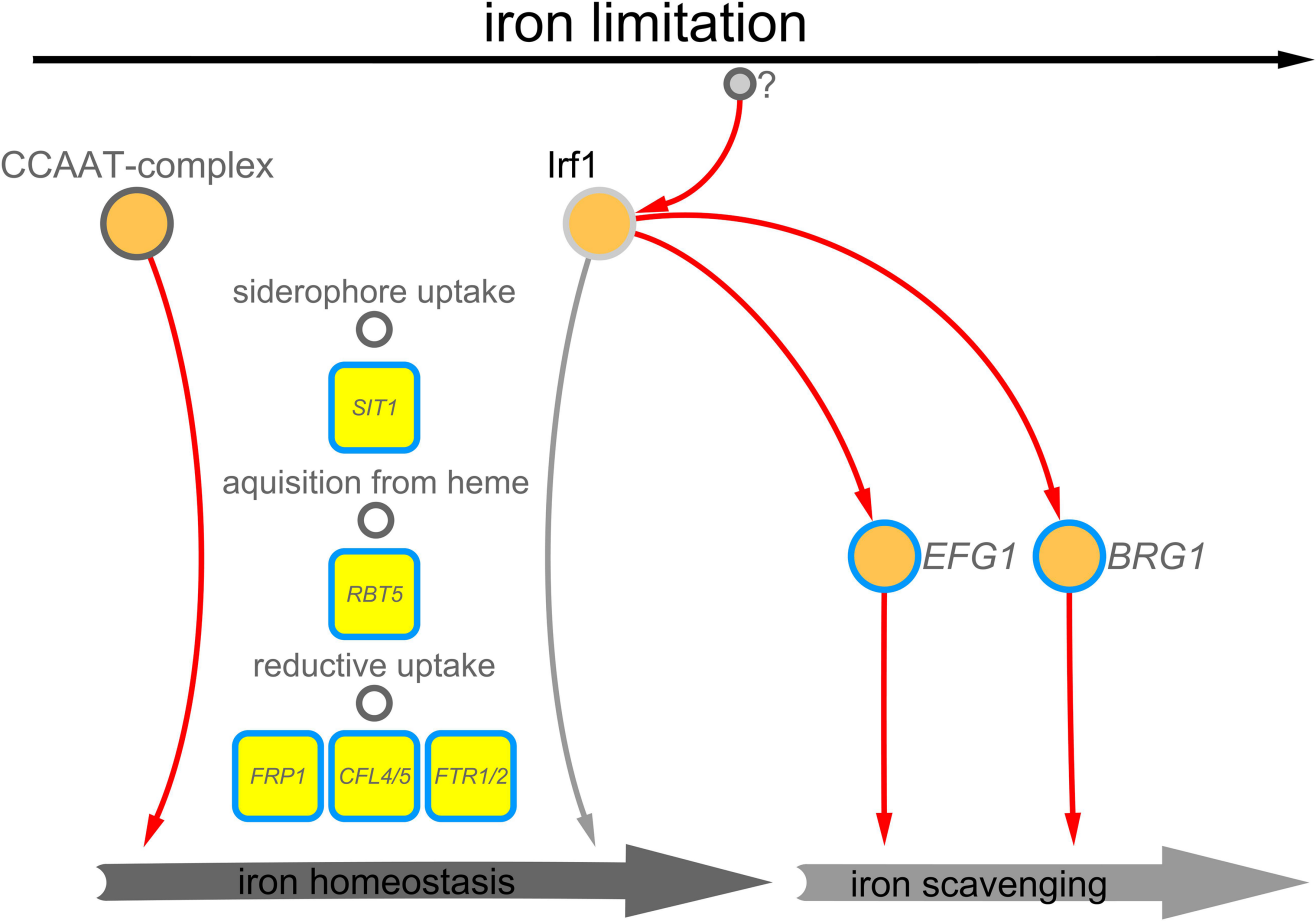
Working model – Irf1 functions at the interface of iron scavenging and iron acquisition. Irf1 is a transcription factor that is activated in iron deficiency, by yet unknown factors, and has a positive influence on the expression of genes that are required for the uptake of iron, but also on genes that are important in the filamentation induced by iron deficiency.

In this study, we have initiated the characterisation of the C_2_H_2_ transcription factor Irf1, previously identified as a potential regulator of hyphal morphogenesis in *C. albicans* (Chauvel et al., 2012). An important aspect for the virulence of *C. albicans* is its ability to reversibly switch between yeast and hyphal cell forms (Jacobsen et al., 2012). Overexpression of *IRF1* resulted in a hyper filamentous phenotype, even under growth conditions favouring yeast growth (Chauvel et al., 2012). In addition to forming true hyphae, *C. albicans* can form pseudohyphae, which essentially resemble the elongated buds of yeast cells and, unlike true hyphae, have a distinct constriction at the septa (Sudbery et al., 2004; Sudbery, 2007). Detailed analysis of the cell morphology revealed that overexpression of *IRF1* initiated the formation of pseudohyphae, whereas the *irf1*-mutant showed no abnormalities except for the formation of cell chains (**Figure 1A**). Moreover, under growth conditions that induce the formation of true hyphae, the *irf1*-mutant showed no defect (**Figure 1B**). We therefore assumed that Irf1 is not involved in the regulation of true hyphae or plays only a minor role. Our current working model describes the activities of the Irf1 transcription factor in *C. albicans* cells stressed by iron deficiency (**Figure 7**). Analysis of the Irf1 gene regulatory network clearly indicates that Irf1 is a positive regulator of iron acquisition and contributes to the maintenance of iron homeostasis in *C. albicans*. Phenotypic analyses support these results, but also indicate that Irf1 is not essential for iron acquisition, in contrast to components of the CCAAT-complex. Rather, the main function of Irf1 under iron-deficient conditions appears to be in the induction of hyphal growth, which could allow the fungus to escape these hostile conditions. By activating *EFG1* and *BRG1*, Irf1 is able to initiate the iron scavenging process. The upstream signalling pathways that lead to the activation of Irf1 during iron deficiency stress are still unknown and waiting to be discovered.

About 20% of all identified Irf1 target genes could be assigned to the gene-ontology category filamentous growth (**Figure 3B**). Among them were positive factors of hyphal formation such as *BRG1*, *EFG1*, *TEC1* and *AHR1*, which are activated by Irf1, as well as the negative hyphal regulator *NRG1*, which is inhibited by Irf1 (**Figures 3B, C**). The two negative hyphal regulators *TCC1* and *RFG1* were also among the direct target genes of Irf1, but they showed no transcriptional regulation upon *IRF1* overexpression; both encode transcription factors that, like *NRG1*, lead to constitutive pseudohyphal growth when inactivated (Braun et al., 2001; Kadosh and Johnson, 2001; Kaneko et al., 2006). However, the transcriptional *IRF1*-hyphal programme differs significantly from known true hyphal programmes. For instance we could not detect enrichment of genes encoding proteins involved in direct processes of hyphal formation, such as septins, components of the polarisome complex or the actin-based cytoskeleton (Sudbery, 2007). Further, except for the activation of *RBT5*, the transcript profile of *IRF1* did not show a signature of a true hyphal programme, as the hyphal-specific genes *HWP1*, *ALS3*, *HGT2*, *HWP1* and *IHD1* (Martin et al., 2013) did not show significant expression changes (**Table S2**, **Figure 2**). Furthermore, the *Candida* toxin-encoding gene *ECE1*, which is also activated during the formation of true hyphae (Birse et al., 1993), even showed significantly decreased expression in the *IRF1* transcript profile (**Figure 3C**); direct binding to the promoter of *ECE1* by Irf1 could not be detected. Thus, Irf1 appears to orchestrate hyphal formation through the regulation of other transcription factors and is therefore upstream in the signalling pathway.

Other identified target genes of Irf1 encode components of the cell wall, in particular the cell wall of hyphae (*SOD4*, *GPM1*, *TEF1*, *YWP1*, *TOS1*, *PGA62*, *RBT5* and *ALS1*) and proteins involved in cell adhesion (*ALS1*, *ALS6*, *ALS9*, *PBR1*, *SDE1*, *EAP1*, *AAF1*), which could explain the observed cell chain phenotype of the *irf1*-mutant. However, a detailed analysis of the cell surface properties is needed to confirm the involvement of Irf1 in these processes.

Interestingly, among the direct target genes of Irf1 were also genes encoding components of the different systems of iron acquisition, for example, *SIT1*, which encodes a transporter of siderophores (Heymann et al., 2002), the haemoglobin receptor *RBT5* (Kuznets et al., 2014) and multiple components of the reductive iron uptake pathway, comprising *FTR1*, *FTR2*, *CFL4* and *CFL5*, *CCC2* and *FET99* (**Figure 3B** and **Figure 4B**). Comparative analysis with the iron deprivation transcriptome revealed that Irf1 contributes to the regulation of a significant subset of these genes and stimulates the expression of genes involved in iron acquisition (**Figure 4B**). Transcript analysis by qPCR under iron deficiency conditions also revealed that Irf1 is involved in the regulation of iron acquisition genes under this condition but is not required for full activation under iron deficiency (**Figure 6A**), which is also in line with the observed mutant phenotype on BPS-containing media (**Figure 5A**). The *IRF1* overexpression strain was able to enhance resistance to BPS, as expected by the strong stimulating effect on iron acquisition genes, but the *irf1*-mutant strain displayed no increased sensitivity as compared to the control strain (**Figure 5A**). Analysis of the co-regulatory network of established iron regulators Sef1, Hap43 and Sfu1 (Chen et al., 2011) indicated that Irf1 might function in parallel to the regulatory circuit, activating expression of iron acquisition genes. No reciprocal regulation between the established iron regulators and Irf1 could be identified. The overexpression of *IRF1* was also able to complement the BPS sensitivity phenotype of a *hap5*-mutant (**Figure 5B**); *HAP5* encodes a subunit of the CCAAT-binding complex, which is essential for growth under iron deficiency conditions (Johnson et al., 2005; Homann et al., 2009). This result demonstrates that Irf1 does not require the CCAAT-complex for transcriptional activation of iron acquisition genes and thus confirms our assumption that Irf1 acts in a parallel signalling pathway.

Another indicator for the involvement of Irf1 in the regulation of iron homeostasis is the observed strong increase in secretion of riboflavin after long-term overexpression of *IRF1* (**Figure 1C**). A direct influence on the production of riboflavin cannot be excluded, since *RIB3*, which codes for a protein of riboflavin biosynthesis (Echt et al., 2004), is a direct target of Irf1. Alternatively, it could be a consequence of iron starvation which induces production of riboflavin through the positive iron acquisition regulator Sef1 (Knight et al., 2002; Demuyser et al., 2020). Surprisingly, both *SEF1* and its antagonist *SFU1* showed increased expression levels at the later time point of *IRF1* overexpression (**Figure 4**), suggesting mis-regulation of the iron sensing and uptake pathway, which could lead to increased production of riboflavin.

Interestingly, Hameed et al. (2008) have shown that iron deficiency can also stimulate hyphal growth of *C. albicans*. Furthermore, genome-wide transcriptome studies of *C. albicans* cells showed that induction of hyphal growth leads to the activation of iron acquisition systems and iron is required for the maintenance of hyphal growth (Martin et al., 2013; Luo et al., 2021). One possible explanation for the correlation is that the fungus requires more iron for hyphal growth, for instance for detoxification of radicals or energy production. Alternatively, this co-regulation could reflect a phenomenon of adaptive prediction, suggesting that hyphal growth and iron deficiency often occur together and that the underlying regulatory structures are therefore tightly interwoven. It was recently shown that the positive iron regulator Sef1 is involved in hyphal growth under embedded conditions (Junier et al., 2022). Moreover, mutants of the CCAAT-complex show defects in their ability to form hyphae (Johnson et al., 2005; Homann et al., 2009), which strengthens the hypothesis that both processes are co-regulated. Our results show that Irf1 is specifically required for hyphal formation induced in iron deficiency (**Figure 6B**). The expression of Irf1-regulated positive hyphal regulators *EFG1*, *AHR1*, *TEC1* and *BRG1* is also activated under iron deficiency conditions, particularly for *EFG1* and *BRG1* (**Figure 6A**). We were able to exclude the involvement of Ahr1 and Tec1 in iron deficiency-induced filamentation, consistently with recently published results on *AHR1* (Henry et al., 2021). In contrast, both the *efg1-* and *brg1*-mutants showed a defect in their ability to form hyphae under iron deficiency (**Figure 6C**, Hameed et al., 2008). Interestingly, *EFG1* and *BRG1* are also required for hyphal formation mediated by *IRF1* overexpression under normal growth conditions, indicating that the Irf1-mediated hyphal programme corresponds to the hyphal programme induced in iron deficiency. Further, it has been shown recently that blocking iron uptake blocks hyphal elongation in a Brg1-dependent manner (Luo et al., 2021). However, the overexpression of *BRG1* results in hyphal growth under iron-deficient conditions, suggesting that Brg1-mediated filamentation can bypass the need of iron. This result can also mean that the fungus does not need additional iron for the formation of filaments, and that iron is only a signalling factor. *C. albicans* is both a commensal and a human pathogen and it can be exposed to very extreme differences in nutrient availability associated with different environments in the human body, which includes iron (Blankenship and Mitchell, 2011). For instance, while the bloodstream is completely iron-depleted, the gastrointestinal tract is an iron-replete environment (Noble, 2013). Accordingly, the fungus has developed a sophisticated control system that allows it to survive under both conditions, to escape both toxic iron concentrations and iron deficiency. The fungus is in constant competition with the host, but also with the other resident microbes. In this context, the functional link between iron uptake and the formation of filaments makes sense, because the fungus can specifically use filamentous growth to escape harsh environmental conditions. Consequently, Irf1-enhanced filamentous growth could represent a nutritional scavenging response, as observed in the budding yeast (Cullen and Sprague, 2012).

The network analysis also revealed an interesting connection between Irf1, the mating network and the recently published commensalism network, whose main components are conserved but whose functions have partially evolved antagonistically (Witchley et al., 2021). While the isolated view of the mating network reveals no apparent function of Irf1, with both positive and negative factors activated by Irf1, the commensalism network reveals that Irf1 binds exclusively to the regulatory sequences of identified inhibitors of commensal growth, comprising *WOR2*, *WOR3*, *WOR4*, *EFG1* and *AHR1*, and accordingly activates their expression (**Figure S4C**). In contrast, no direct binding to the regulatory sequences of *WOR1*, *CZF1* and *SSN6*, which promote commensal growth, could be detected (**Figure S4C**). This is particularly interesting in view of the antagonistic function of Irf1 in mammalian gut colonisation and systemic infection; the study by Perez et al. (2013) suggested that Irf1 (Orf19.217) is an inhibitor of commensal growth, but in contrast is required for the expression of the full virulence potential of the fungus. It can also be speculated that Irf1 inhibits commensal growth in the gut by stimulating hyphal growth. Similar dynamics were observed for transcription factors that promote hyphal growth, including Irf1 direct target genes *EFG1*, *BRG1* and *TEC1*, as the corresponding mutants exhibited increased fitness in commensalism models (Pierce and Kumamoto, 2012; Pande et al., 2013; Witchley et al., 2019). Further, a high number of identified genes regulated by Irf1 are also transcriptionally modulated during commensal growth of *C. albicans* cells in the GI tract (**Figure S4A**) suggesting a functional link between the two processes. The transcription factor Irf1 thus appears to play a role in the regulation of *C. albicans* growth behaviour in the host. Since the main regulators of the commensalism network are also intertwined with the mating network, it is not surprising that the *irf1*-mutant exhibits a reduced white-to-opaque switching rate (Lohse et al., 2016), which presumably results from a mis-regulation of its network components.

Overall, our study highlights the importance of maintaining trace metal homeostasis and regulating morphogenesis during commensalism and pathogenicity of *C. albicans* and shows once more that both features are intertwined. The transcription factor Irf1 likely underwent functional rewiring during coevolution with the human host to facilitate growth of *C. albicans* in this complex environment.

## Data availability statement

The original contributions presented in the study are included in the article/**Supplementary Material**. Further inquiries can be directed to the corresponding author/s.

## Author contributions

LW constructed the strains and performed all experiments. LW performed the bioinformatics analyses of ChIP-Seq and RNA-Seq derived data and the network construction. LW and SZ performed GO analyses and gene-set enrichment analyses. LW, SZ, AH-C, VB, SB-B, and Cd’E designed the experiments and contributed to writing of the manuscript. All authors contributed to the article and approved the submitted version.

## Funding

The work was supported by the Agence Nationale de la Recherche grant (CANDIHUB, ANR-14-CE-0018). SZ is a Pasteur Network Affiliate Program Fellow. Work in the laboratory of Cd’E is supported by the French Government’s Investissement d’Avenir program (Laboratoire d’Excellence Integrative Biology of Emerging Infectious Diseases, ANR-10-LABX-62-IBEID). AHC was the recipient of a post‐doctoral fellowship from the European Commission (ERA‐Net Infect‐ERA, FUNCOMPATH, ANR‐14‐IFEC‐0004), from the French Government’s Investissement d’Avenir program (Institut de Recherche Technologique BIOASTER, ANR‐10‐AIRT‐03) and from the National Council of Science and Technology, Mexico. VB was supported by a grant from the Pasteur-Paris University (PPU) International PhD program and the “Fondation Daniel et Nina Carasso”.

## Acknowledgments

We thank all members of the Unité Biologie et Pathogénicité Fongiques, particularly Laxmi Rai and Thierry Mourer, for their support and numerous insights during the course of this project. We are also thankful to Juliana Pipoli Da Fonseca for supporting our ChIP-Seq and RNA-Seq experiments at the Biomics platform at Institut Pasteur and to Gaëlle Lelandais for sharing her insights into NGS derived data analysis.

## Conflict of interest

The authors declare that the research was conducted in the absence of any commercial or financial relationships that could be construed as a potential conflict of interest.

## Publisher’s note

All claims expressed in this article are solely those of the authors and do not necessarily represent those of their affiliated organizations, or those of the publisher, the editors and the reviewers. Any product that may be evaluated in this article, or claim that may be made by its manufacturer, is not guaranteed or endorsed by the publisher.

## Supplementary material

Supplementary Table 1 Strains and oligonucleotides used in this study.

Supplementary Table 2 Summary of transcriptomic and ChIP-Seq analyses.

Supplementary Figure 1 Protein sequence alignment of Irf1 and Cmr3. Amino acid sequences of *C. albicans* Irf1 and *S. cerevisiae* Cmr3 were obtained from candidagenome.org and yeastgenome.org. The sequence alignment was performed with Clustal Omega version 1.2.4 (Goujon et al., 2010). The zinc finger encoding region is highlighted by orange underlining.

Supplementary Figure 2 Expression of genes involved in riboflavin synthesis. Heatmap of expression differences (log2 fold-changes) of genes involved in heme binding, heme biosynthesis, expression of flavodoxin-like proteins or riboflavin biosynthesis (* indicates that the gene is a direct target of Irf1).

Supplementary Figure 3 Iron co-regulatory network. Venn diagram showing number of shared target genes among Irf1, Hap43, Sef1 and Sfu1. The corresponding target genes of the respective transcription factors were obtained from (http://pathoyeastract.org/).

Supplementary Figure 4 Irf1 is central to networks governing mating and commensalism of *C. albicans*. **(A)** Transcriptome data of *C. albicans* cells grown in the GI tract of mice were taken from Rosenbach et al. (2010) and compared with the transcriptome data of *C. albicans* overexpressing *IRF1* from the tetracycline inducible TET promoter in YPD medium. **(B)** Comparison of Irf1 direct target genes obtained by ChIP-Seq with the transcriptome of *C. albicans* grown in the GI tract of mice. Genes whose names are highlighted also show a significant difference in expression when *IRF1* is overexpressed (blue = down, orange = up, black = no significant change). **(C)** Position of Irf1 in the mating and the commensalism core network. Associations are based on networks illustrated in Witchley et al. (2021) and ChIP-chip/ChIP-Seq data from previous publications summarized in *DataSheet_1 Transcriptional Circuits Regulating Developmental Processes in Candida albicans* taken from Rodriguez et al. (2020). Red edges indicate activation, black edges repression and grey edges indicate that no change in expression was observed. Of note, Irf1 only binds to negative regulators of commensalism.

Supplementary Figure 5 Transcriptional control of Irf1 under iron starvation. Based on the relative transcript levels shown in , fold changes of the indicated genes were calculated, comparing treated with untreated growth conditions (+BPS/-BPS). Asterisks indicate significant differences in gene expression between the two conditions determined by a two-tailed Student’s *t*-test. (*) *P* < 0.05; (**) *P* < 0.01; (***) *P*<0.001.
