# Supplementary figures and images for: Functional Portrait of Irf1 (Orf19.217), a Regulator of Morphogenesis and Iron Homeostasis in Candida albicans
Source: Front Cell Infect Microbiol. 2022 Aug 8;12:960884. doi: 10.3389/fcimb.2022.960884 (PMC9393397; doi:10.3389/fcimb.2022.960884)

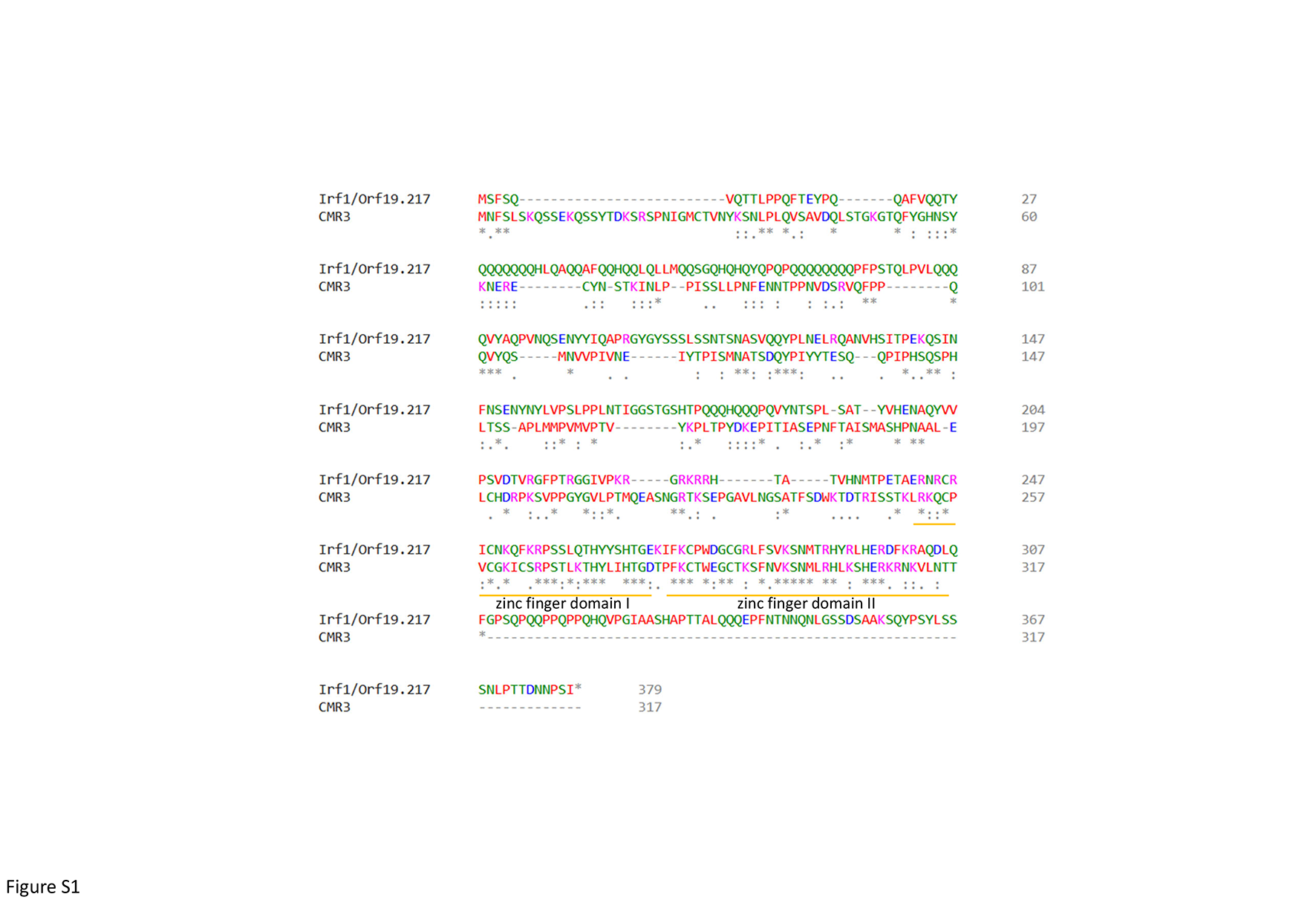

Supplement: Supplementary Figure 1 — Protein sequence alignment of Irf1 and Cmr3. Amino acid sequences of C. albicans Irf1 and S. cerevisiae Cmr3 were obtained from candidagenome.org and yeastgenome.org. The sequence alignment was performed with Clustal Omega version 1.2.4 (Goujon et al., 2010). The zinc finger encoding region is highlighted by orange underlining. [file Image_1.tif]

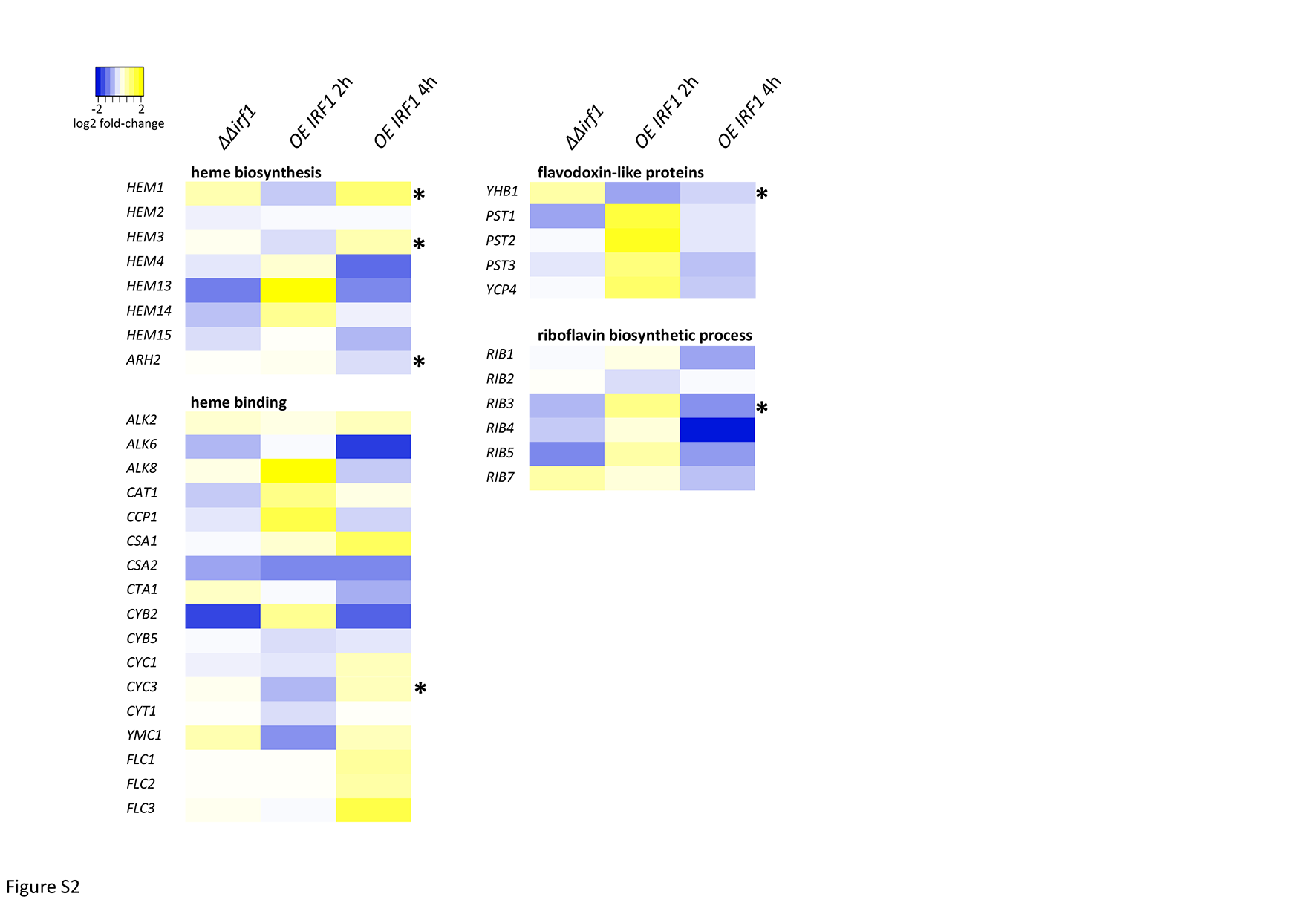

Supplement: Supplementary Figure 2 — Expression of genes involved in riboflavin synthesis. Heatmap of expression differences (log2 fold-changes) of genes involved in heme binding, heme biosynthesis, expression of flavodoxin-like proteins or riboflavin biosynthesis (* indicates that the gene is a direct target of Irf1). [file Image_2.tif]

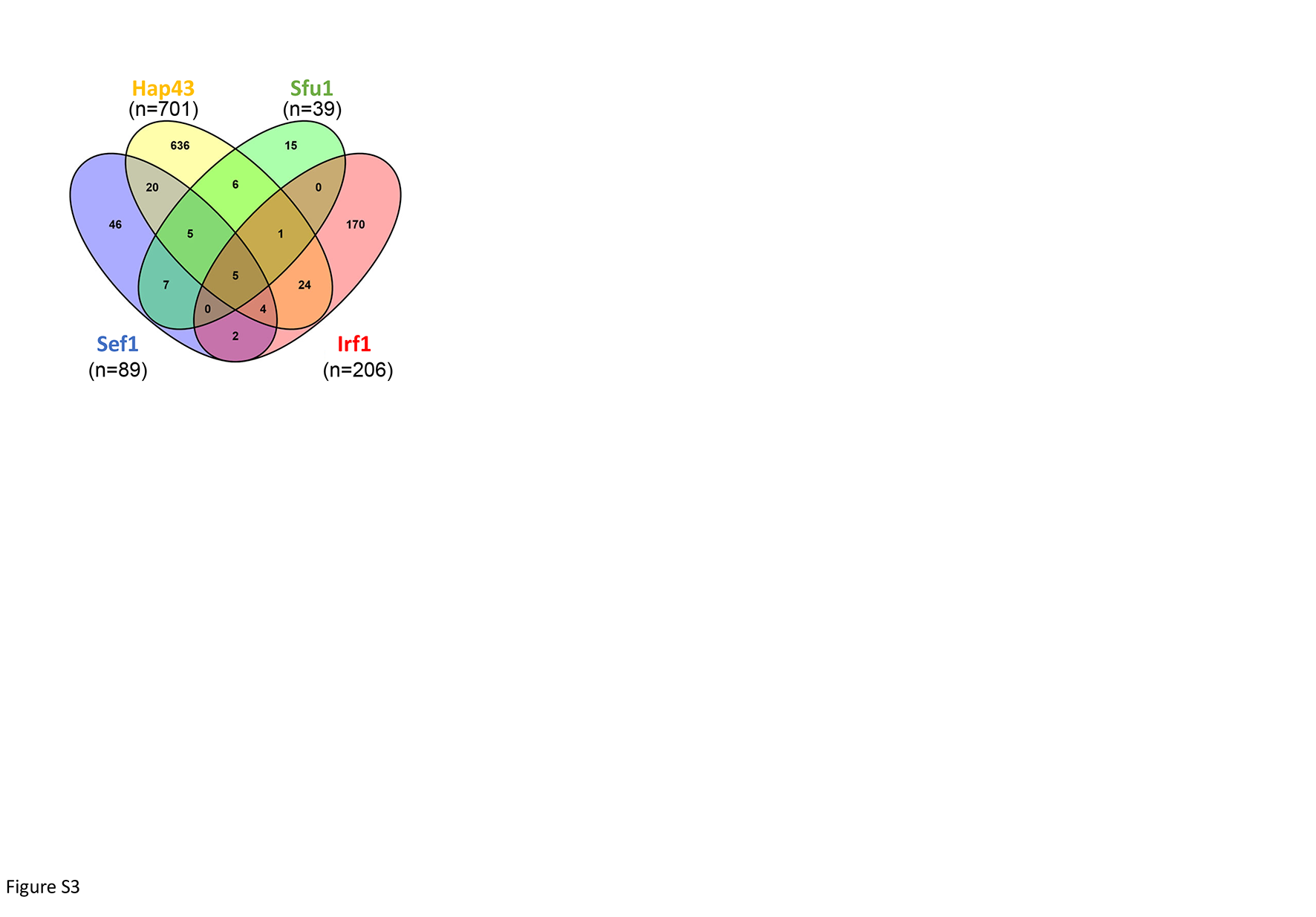

Supplement: Supplementary Figure 3 — Iron co-regulatory network. Venn diagram showing number of shared target genes among Irf1, Hap43, Sef1 and Sfu1. The corresponding target genes of the respective transcription factors were obtained from (http://pathoyeastract.org/). [file Image_3.tif]

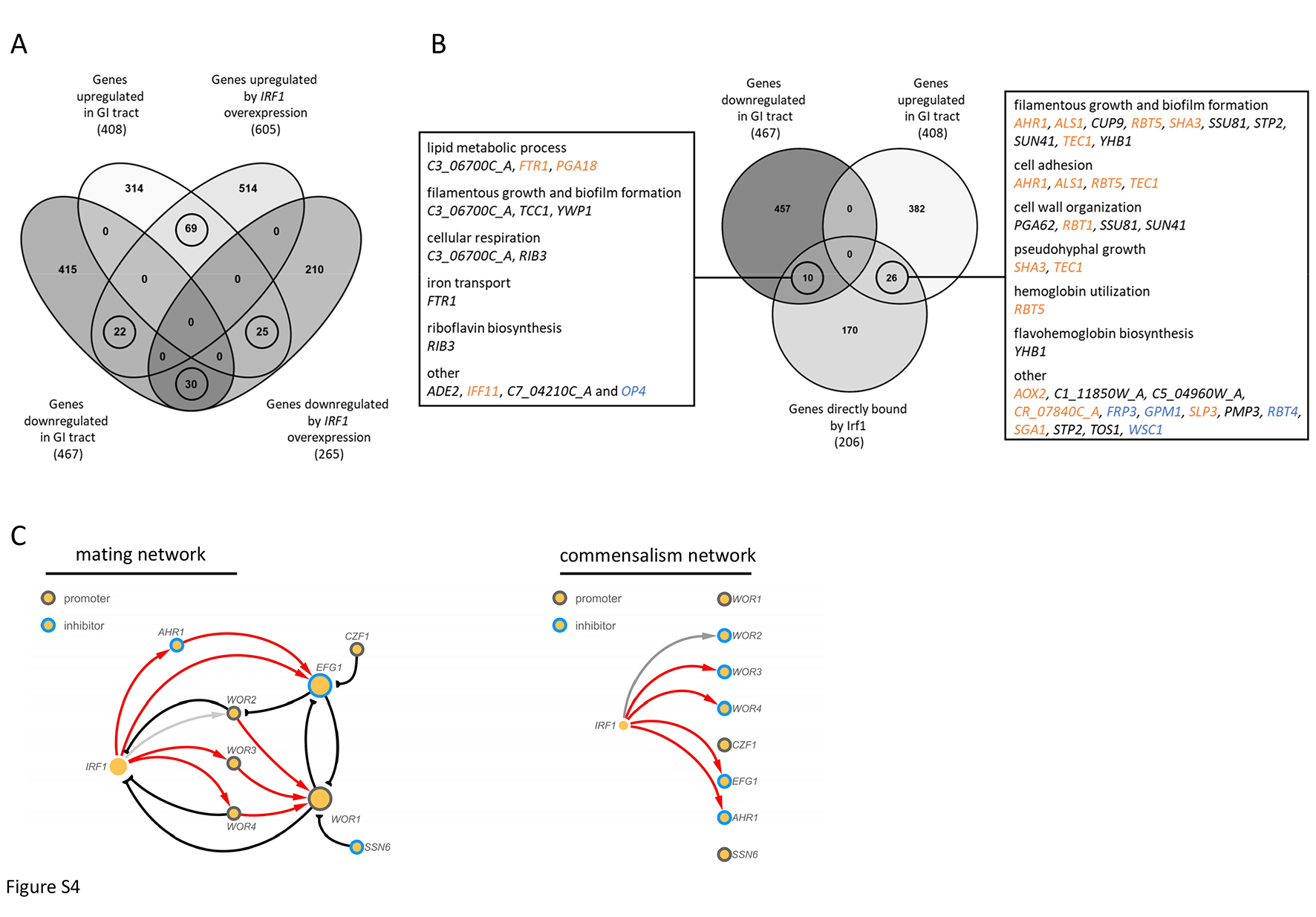

Supplement: Supplementary Figure 4 — Irf1 is central to networks governing mating and commensalism of C. albicans. (A) Transcriptome data of C. albicans cells grown in the GI tract of mice were taken from Rosenbach et al. (2010) and compared with the transcriptome data of C. albicans overexpressing IRF1 from the tetracycline inducible TET promoter in YPD medium. (B) Comparison of Irf1 direct target genes obtained by ChIP-Seq with the transcriptome of C. albicans grown in the GI tract of mice. Genes whose names are highlighted also show a significant difference in expression when IRF1 is overexpressed (blue = down, orange = up, black = no significant change). (C) Position of Irf1 in the mating and the commensalism core network. Associations are based on networks illustrated in Witchley et al. (2021) and ChIP-chip/ChIP-Seq data from previous publications summarized in DataSheet_1 Transcriptional Circuits Regulating Developmental Processes in Candida albicans taken from Rodriguez et al. (2020). Red edges indicate activation, black edges repression and grey edges indicate that no change in expression was observed. Of note, Irf1 only binds to negative regulators of commensalism. [file Image_4.tif]

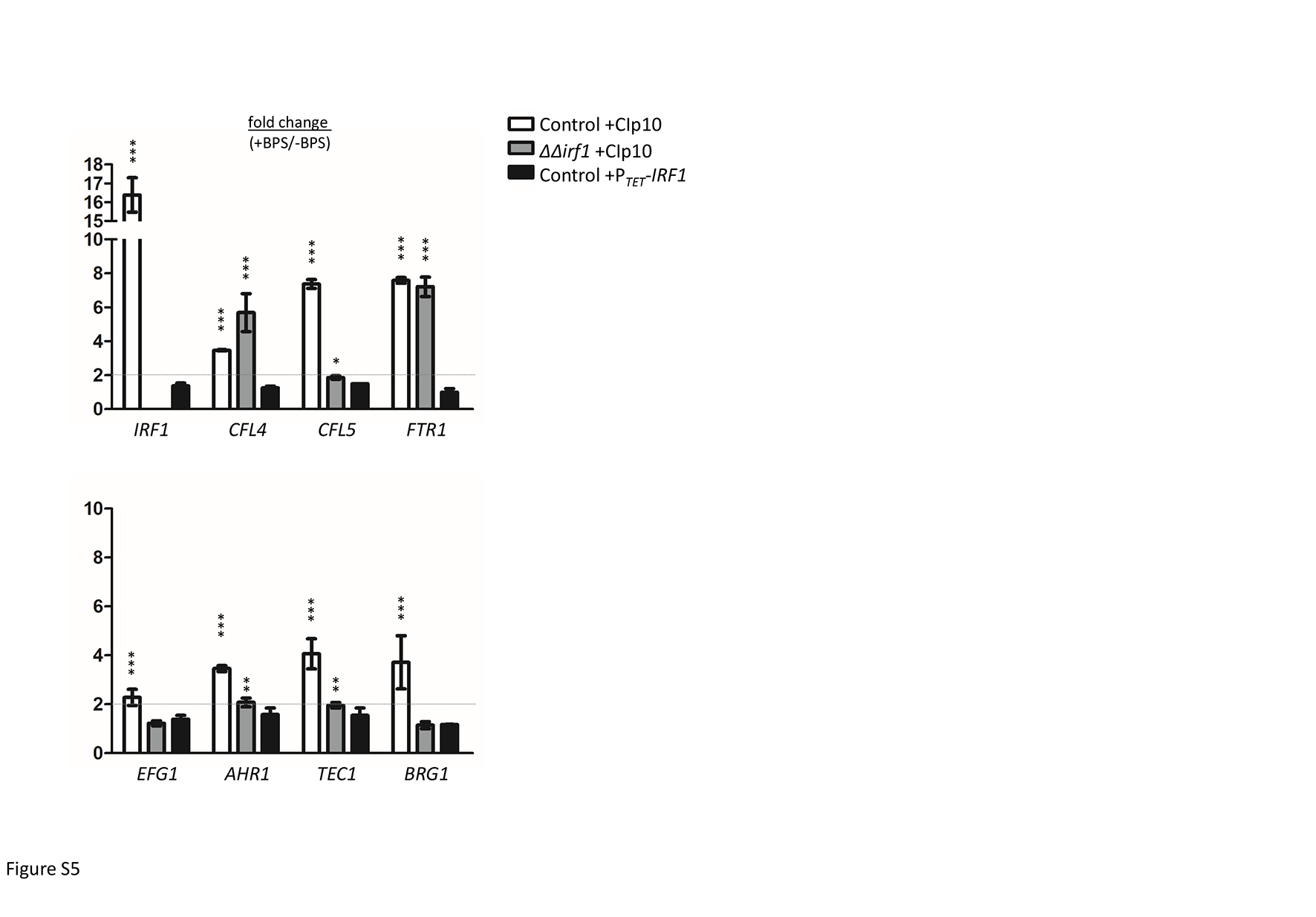

Supplement: Supplementary Figure 5 — Transcriptional control of Irf1 under iron starvation. Based on the relative transcript levels shown in , fold changes of the indicated genes were calculated, comparing treated with untreated growth conditions (+BPS/-BPS). Asterisks indicate significant differences in gene expression between the two conditions determined by a two-tailed Student’s t-test. (*) P < 0.05; (**) P < 0.01; (***) P<0.001. [file Image_5.tif]
